# Successful ceritinib treatment in a man with MPE and an ALK fusion gene mutation after multiple treatments

**DOI:** 10.1186/s40064-016-3674-3

**Published:** 2016-12-07

**Authors:** Hangping Wei, Fangming Du, Yifang Lu, Juan Wei, Xiaofang Dong

**Affiliations:** Department of Medical Oncology, Dongyang People’s Hospital, No. 60 West Wuning Road, Dongyang, 322100 Zhejiang People’s Republic of China

**Keywords:** Ceritinib, Malignant pleural effusion, Lung adenocarcinoma, ALK fusion gene mutation

## Abstract

**Introduction:**

Ceritinib is a second-generation anaplastic lymphoma kinase (ALK) inhibitor. It inhibits two of the most common ALK-mutants that confer resistance to crizotinib. Ceritinib was approved by Food and Drug Administration in April 2014. However, the efficacy of ceritinib in Asian patients have not been widely studied. Decrease of malignant pleural effusion (MPE) has been rarely reported after treatment with ceritinib.

**Case description:**

A 50-year old man diagnosed with stage IV lung adenocarcinoma presented with MPE and an ALK fusion gene mutation. The patient showed partial response to ceritinib after 2-month treatment. Ultrasound showed MPE significantly decreased.

**Discussion and evaluation:**

Ceritinib is a good choice, as a targeted therapy, which is more prospect in the advanced cancer patients than the traditional therapy.

**Conclusion:**

Ceritinib seems to have a good efficacy in reducing MPE in advanced Asian lung adenocarcinoma patients, when other chemotherapy failed.

## Background

During the past decades, platinum-based chemotherapy doublets (e.g. cisplatin/pemetrexed and carboplatin/gemcitabine) was once the standard therapy for non-small-cell lung cancer (NSCLC) (Schiller et al. 2002). In recent years, treatment paradigm has been changed by identification of activating mutations within the epidermal growth factor receptor (EGFR) to EGFR tyrosine kinase inhibitors (EGFR-TKIs). More recently, ALK translocation was described as a oncogenic driver in NSCL (Kaczmar and Mehra 2015; Vansteenkiste 2014). Both echinoderm microtubule-associated protein like 4-anaplastic lymphoma kinase and ALK locate on chromosome 2. The EML4-ALK fusion gene was found in 3–7% of NSCLC patients, who are crizotinib-sensitive. Crizotinib is an oral small-molecule TKI that targets ALK, which has received accelerated approval from the United States FDA (Shaw et al. 2014). However, resistance to crizotinib often occurs after approximately 8-month treatment (Muller et al. 2016). Ceritinib is the second generation of ALK inhibitors approved by FDA in April 2014. It inhibits two of the most common ALK-mutants that confer resistance to crizotinib: L1196M and G1269A, and other resistance mutations are C1156Y, S1206Y, 1151Tins and G1202R, for example (Shaw et al. 2016). Ceritinib is 20 times more potent than crizotinib and has demonstrated clinical efficacy in patients with ALK-positive NSCLC who have failed therapy with crizotinib (Muller et al. 2016). With these breakthrough discoveries, targeted treatment is now widely used in clinical practice, which is more effective and has less adverse effect than those standard chemotherapy (Solomon et al. 2014; Qian et al. 2014). Some study showed that ceritinib provided clinically meaningful and durable responses with manageable tolerability in chemotherapy- and crizotinib-pretreated patients (Nishio et al. 2015; Crino et al. 2016). However, the drug is not still in clinical trials in Asian population and the efficacy of reduce against MPE has barely be found. We present a case report of a 50-year-old man with MPE who is stage IV lung adenocarcinoma and an ALK mutation after other multiple treatments showed a good response to ceritinib. Nearly 1 month later, the symptoms such as chest distress and tachypnea completely disappeared. Two months later, ultrasound showed that MPE significantly decreased. The patient demonstrated a partial response (PR).

## Case presentation

**Fig. 1:**
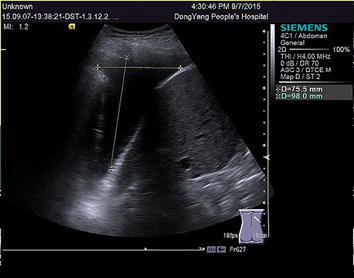
Ultrasound on September 7 2015 showed that the diameter of anteroposterior and vertical respectively was 98 and 76 mm in the *right*, and 33 and 71 mm in the *left* (1 month later)

**Fig. 2:**
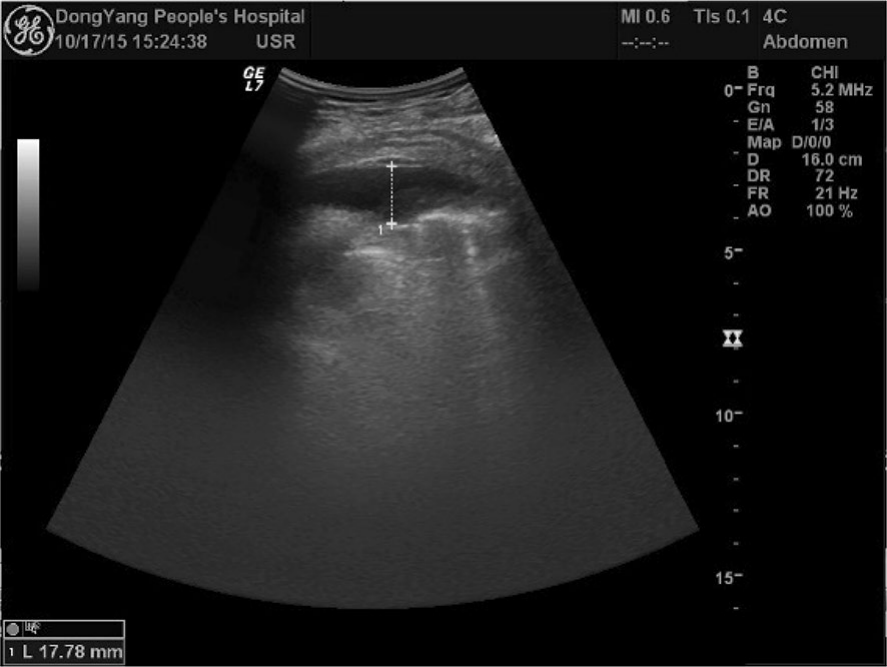
Ultrasound on October 17 indicated that bilateral bulk pleural effusion was almost completely disappeared (2 months later)

**Fig. 3:**
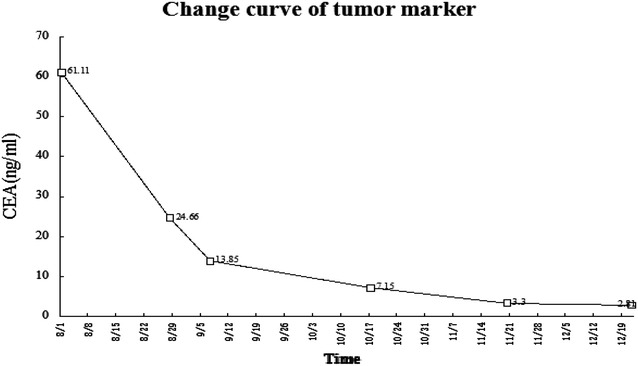
Curve of CEA level during the treatment

**Fig. 4:**
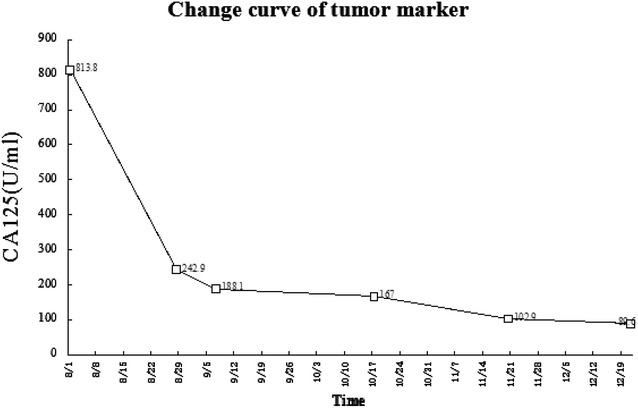
Curve of CA125 level during the treatment

Nearly 4 years ago, a 47-year-old man presented with a 2-month history of cough and sputum. A chest computerized tomography (CT) revealed a pulmonary mass in the right lung, and pleural and bilateral pulmonary carcinomatosis. The biopsy showed that the pulmonary mass was adenocarcinoma. Further confirmed by a PET-CT, the patient was diagnosed with lung adenocarcinoma with multiple metastases (cTxNxM1, Stage IV), involving the contralateral lung, bone, adrenal gland and lymph node. Molecular testing found the patient was mutation-type of ALK translocation. The patient is asymptomatic and with a stable general condition. The patient was treated with six-course chemotherapy of cisplatin/pemetrexed. PR showed after the first two-course treatment with RECIST1.1 criteria. One year later, the patient showed pulmonary progression and started to receive crizotinib, which achieved stable disease (SD) as the maximum response, accoeding to the RECIST1.1 criteria. After 1 year, magnetic resonance imaging (MRI) revealed brain metastases. So the patient initiated whole brain radiotherapy combination with temozolomide (150 mg m^−2^, 28 days for a cycle), followed by the other chemotherapy with two-course of carboplatin and gemcitabine to consolidate the curative effect and to stability the lung lesions, using SD as the maximum response with RECIST1.1 criteria. Six months later, the patient showed pulmonary progression and initiated the other chemotherapy with four-course of cisplatin/pemetrexed, with SD as the response, according to the RECIST1.1 criteria. Eight months later, the patient, a 51-year-old man, showed clinical progression with the symptoms such as serious chest distress and tachypnea. Ultrasound revealed bilateral bulk pleural effusion. The right diameter of anteroposterior and vertical were respectively 127 and 130 mm, and in the left were 89 and 94 mm. Due to the poor general condition (ECOG PS is three point), ceritinib, buyed from Hong Kong, 750 mg once daily was started. One month later, symptoms like chest distress and tachypnea completely disappeared. Ultrasound showed that pleural effusion significantly reduced (Fig. 1). The diameter of anteroposterior and vertical respectively were 98 and 76 mm in the right, and 33 and 71 mm in the left. Two months later, ultrasound revealed small amount of pleural effusion, indicating that bulk pleural effusion was nearly completely disappeared (Fig. 2), with II degree of nausea. During the treatment process, selected tumor markers were also significantly reduced, which were summarized in Table 1. Change curve of tumor marker (CEA and CA125) was presented in Figs. 3 and 4. Currently, the patient demonstrated a PR, according to Response Evaluation Criteria in Solid Tumors (RECIST 1.1), and have a very good general status (Karnofsky 80–90%).

**Table 1:** The tumor markers were significantly reduced during the treatment progress

Time	8/1	8/28	9/7	10/17	11/20	12/21
CEA (ng/ml)	61.11	24.66	13.85	7.15	3.3	2.81
CA125 (U/ml)	813.8	242.9	188.1	167	102.9	89.6

## Discussion

 Discovery of oncogenic drivers has revolutionized management strategy for various cancer patients (Sereno et al. 2015). Moreover, development of personalized medicine of lung adenocarcinoma, the discovery of various genetic alterations that promote cancer growth and survival, such as EGFR mutations and EML4-ALK rearrangements, have revolutionized treatment paradigms for patients (Kwak et al. 2010). Targeted therapies significantly increase disease-free survival (DFS) and overall survival (OS) compared to conventional chemotherapy such as platinum-based chemotherapy. Chromosomal rearrangements of a ALK were detected in 1.6–8.6% of unselected NSCLC patients (Scagliotti et al. 2012; Forde and Rudin 2012). Crizotinib, a druggable ALK receptor tyrosine kinase for cancer treatment, have been approved for the treatment of metastatic NSCLC, with marked improvement of progression-free survival of patients (Chan et al. 2016). A subsequent clinical trial established crizotinib as the first-line treatment for ALK positive NSCLC. The PFS of crizotinib is 10.9 months while patients using standard platinum doublet therapy with either cisplatin or carboplatin and pemetrexed only achieved 7.0 months (Solomon et al. 2014). Unfortunately, crizotinib resistance inevitably occurred. In general, mechanisms of acquired drug resistance could be classified into two main categories. The target gene itself could be altered either by mutation or by amplification. On the other hand, tumor cells might lose their dependency from the inhibited signaling pathway by activating alternative signaling pathways (Rothschild and Gautschi 2013). In up to one-third of relapsing patients, crizotinib resistance is mediated by secondary resistance mutations located in the ALK tyrosine kinase domain. The most commonly identified resistance mutation is the gatekeeper mutation L1196M and G1269A (Solomon et al. 2014). Thanks to better understanding of resistance mechanisms to crizotinib, new therapeutic approaches have been developed. The development of acquired resistance, however, poses a serious clinical challenge.

Ceritinib (LDK378, Zykadia^®^, Novartis Pharmaceuticals, Basel, Switzerland) is an oral, small-molecule, ATP-competitive, TKI of ALK. In preclinical trials, ceritinib seems to be a more potent ALK inhibitor than crizotinib (Doebele et al. 2012). Furthermore, ceritinib is able to overcome resistance to crizotinib. The US FDA granted accelerated approval to ceritinib in April 2014 based on several promising clinical trials mentioned below. Shaw et al. reported landmark ASCEND-1 study in March 2014. Among 114 patients, 1 achieved complete response, 65 partial responses (57%) and 25 (22%) instances of stable disease. In addition, two clinical trials have provide ceritinib have meaningful and durable responses in Asian patients (Nishio et al. 2015; Crino et al. 2016). In this case, patient achieved the second PR, after the first chemotherapy, while others with SD. MPE and tumor markers were significantly reduced. It is worth mentioned that score of Questionnaire of Quality of Life (QQL) in this case was significant improved. It is more important in the advanced cancer patients than the evaluation index such as PFS or OS. There was a point to dose about ceritinib in Asian people. The body surface area of the patient is 1.9, similar to that of the Americans’ but larger than the average of Asian people. The above two clinical trials, Ceritinib maximum-tolerated dose was 750 mg once daily in Asian patients (Nishio et al. 2015; Crino et al. 2016). Of course, it should be noted that studies with larger samples and the doses should be performed to get more accurate results.

In this case, it should also be noticed that targeted therapy is a good choice, after multiple treatments. So, targeted therapy is the trend of the development of lung cancer. However, two clinically validated and FDA approved lung cancer predictive biomarkers (EGFR and ALK translocations) occur in only about 20% of lung adenocarcinomas and acquired resistance develops to first generation drugs (Cagle et al. 2016). More biomarkers and targeted drugs should be found. Maybe newer ALK-TKIs like ASP3026, AP26113, and X-396 will be of benefit for these patients.

## Conclusion

This patient showed a quick and significant response to ceritinib during 2 months and a marked reduction of MPE and tumor marker a few days after starting ceritinib. It suggests that ceritinib have a good efficacy against MPE in advanced lung adenocarcinoma in Asian Patients, even if failed with other multimodal treatments.

## Abbreviations

EML4-ALK: echinoderm microtubule-associated protein like 4-anaplastic lymphoma kinase
FDA: food and drug administration
MPE: malignant pleural effusion
NSCLC: non-small-cell lung cancer
EGFR: epidermal growth factor receptor
EGFR-TKIs: epidermal growth factor receptor-tyrosine kinase inhibitors
PR: partial response
SD: stable disease
DFS: disease-free survival
OS: overall survival
QQL: questionnaire of quality of life
